# Effect of Film Morphology on Electrical Conductivity of PEDOT:PSS

**DOI:** 10.3390/nano14010095

**Published:** 2023-12-29

**Authors:** Aditya Saha, Daisuke Ohori, Takahiko Sasaki, Keisuke Itoh, Ryuji Oshima, Seiji Samukawa

**Affiliations:** 1 Institute of Fluid Science, Tohoku University, Sendai 980-8577, Japan; saha.aditya.q6@dc.tohoku.ac.jp (A.S.);; 2 Institute for Materials Research, Tohoku University, Sendai 980-8577, Japan; takahiko.sasaki.d3@tohoku.ac.jp; 3 Industrial Technology Institute, Miyagi Prefectural Government, Sendai 980-0014, Japan; ito-ke592@pref.miyagi.lg.jp; 4 National Institute of Advanced Industrial Science and Technology, Tsukuba 305-8560, Japan; r.oshima@aist.go.jp; 5 Institute of Communications Engineering, National Yang Ming Chiao Tung University, Hsinchu City 30010, Taiwan

**Keywords:** PEDOT:PSS, morphology, conductivity

## Abstract

Commercially available formulations of the popular conductive polymer, poly(3,4-ethylenedioxythiophene):polystyrene sulfonate (PEDOT:PSS) are aqueous dispersions that require the addition of secondary dopants such as dimethyl sulphoxide (DMSO) or ethylene glycol (EG) for fabricated films to have the desired levels of conductivity. Clevios^TM^ F HC Solar, a formulation of PEDOT:PSS produced by Heraeus, GmbH, achieves over 500 S/cm without these secondary dopants. This work studies whether secondary dopants such as DMSO have any additional effect on this type of PEDOT:PSS. The temperature dependencies of the conductivity of F HC Solar spin-coated thin films measured using a four-probe method seem to exhibit different charge transport properties compared with secondary doped PH1000. Observations made using atomic force microscopy (AFM) show that different concentrations of DMSO affect the orientation of the PEDOT domains in the thin film. These morphological changes cause room temperature conductivity to reduce from 640 S/cm in pristine films to as low as 555 S/cm after adding 7 wt% of DMSO along the film. Such tuning may prove useful in future applications of PEDOT:PSS, such as nanoprobes, transistors and hybrid solar cells.

## 1. Introduction

In recent years, the tremendous technological progress in nanoelectronics has fostered the development of organic conjugated polymers, which have emerged as a promising class of materials to engineer many devices from electrochemical transistors, bioelectronic devices, and nanoprobes. At the same time, research on inexpensive and renewable energy devices has become a generational challenge due to the alarming rate and effects of global warming [1]. Photovoltaic (PV) technology has advanced to the point that it is projected to supply a large fraction of the world’s energy demand in the future [2]. However, in spite of falling prices, the cost of the PV module still accounts for the largest percentage of investments in solar cells [3]. Contributing factors to this situation are the use of scarce resources, such as indium and gallium, and high temperature (≥850 °C) dopant diffusion and annealing processes that are usually required in the manufacture of silicon homojunction photovoltaic cells, which dominate the market. Replacing these materials and processes with simpler earth-abundant counterparts and low-temperature techniques is the goal of the current so-called third-generation PV devices. Commercially available poly(3,4-ethylenedioxythiophene):poly styrenesulfonate (PEDOT:PSS) is one of the best choices for making such devices, owing to its high transmittance in the visible light range, high adjustable conductivity, intrinsic high work function, excellent thermal stability, and good film forming capability [4,5,6,7]. It has attracted a growing amount of research into silicon-organic hybrid solar cells (SOHCs) in various configurations [8,9,10,11,12]. Consequently, architectures that use *n*-type Si as the absorber and PEDOT:PSS as the front transparent carrier-selective contact have thus far been able to achieve device efficiencies exceeding 17% [13].

**Figure 1:**
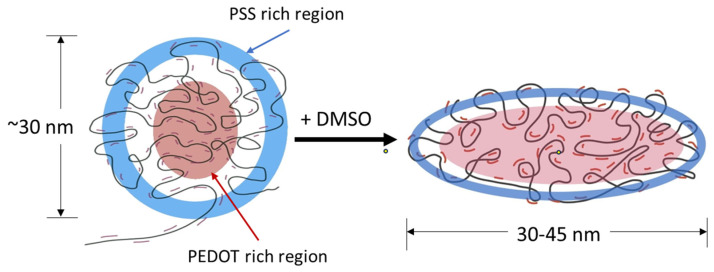
Graphical representation of the PEDOT:PSS nanostructure. PEDOT chains (red) sparingly attached to much longer PSS chains (black) forming a spherical gel particle with PSS-rich regions (blue) forming an outer shell and the PEDOT-rich regions (red) making up the core [15]. Upon deposition, these PEDOT:PSS particles are usually observed as elongated grains [23].

PEDOT:PSS aqueous dispersions were first commercialized under the trade name Baytron^®^ by Bayer AG, followed by H.C. Starck, GmbH, and most recently by Heraeus, GmbH, under the trade name Clevios^TM^ [14]. PEDOT:PSS is a complex polyelectrolyte chain, where PEDOT-rich sections usually migrate towards the center, while the PSS-rich portions form a shell-like structure due to their hydrophilic nature (Figure 1). Most PEDOT:PSS dispersions contain 30 nm colloidal nanoparticles. In what follows, these nanoparticles will be referred to as gel particles when they are in their dispersed state. The morphology of these gel particles was recently studied in detail by Jain et al. [15]. PEDOT:PSS dispersions such as Clevios PH500, PH750, or PH1000 usually need the addition of a high boiling point and polar organic solvents, such as dimethyl sulphoxide (DMSO), sorbitol, or ethylene glycol (EG), to reach high conductivities [16,17,18,19]. PEDOT:PSS gel particles in solution, when deposited, form a flat, pancake-like microstructure (Figure 1). The size of the structures may be controlled through the addition of these solvents during the preparation of the solution or post-treatment with the goal of transforming the compact PEDOT coils into more elongated shapes [20,21]. Studies have shown that mixing these solvents and annealing at higher temperatures change the morphology of PEDOT:PSS films and consequently improve their conductivity [22,23,24,25,26,27]. It has also been observed that the morphology of the PEDOT:PSS nanostructures significantly affects the bulk transport properties [28,29].

Recently, Heraeus has distributed a PEDOT:PSS dispersion named F HC Solar that advertises 500 S/cm without any secondary dopant. This makes it a good candidate for organophotovoltaics without the need for any pre-treatment [30]. Unfortunately, specifics regarding the solution, such as the PEDOT content, or the presence of additives, have not been revealed. While the electrical characteristics and morphology of PEDOT:PSS have been widely discussed, not much information exists for F HC Solar. This may probably be due to the assumption that they are comparable. The behavior of this solution in the previously mentioned solvents is not known to the best of our knowledge. In this research, we show that the charge mobility of Clevios F HC Solar (hereafter referred to as PEDOT-F) thin films is likely very different from Clevios PH1000 (hereafter referred to as PEDOT-P), which is a widely used PEDOT:PSS dispersion, also made by Heraeus. Additionally, PEDOT-F films were also doped with commonly added wt% of DMSO to check whether a large increase in conductivity would be observed, as in the case of PEDOT-P.

## 2. Materials and Methods

In this study, 10 mm × 10 mm, 270 µm thick silicon wafers with resistivities greater than 10,000 Ω.cm, manufactured using the Czochralski (CZ) method, were cleaned with piranha solution (2:1 by volume of H_2_SO_4_ and H_2_O_2_, respectively) to remove dust and organic residue. The native silicon oxide layer on the substrates was etched away using a 5% HF solution for 2 min. These were then treated with neutral beam oxidation (NBO) for 400 s at 500 W bias and at a substrate temperature of 400 °C [31]. This NBO is a low-damage technique in which oxygen plasma is passed through a high aspect ratio carbon aperture onto the substrate. The aperture blocks all high-energy UV radiation and neutralizes all charged species in the plasma, leaving only neutral hyperthermal (<10 eV) oxygen radicals, which are absorbed by the silicon surface in a damage-free process. This results in a passivated and highly pure silicon dioxide surface with a roughness of 0.57 nm, making for a better reference substrate layer than native silicon oxide. The PEDOT-P (Clevios PH1000 from made by Heraeus in Leverkusen, Germany) and PEDOT-F (F HC Solar, also made by Heraeus) aqueous dispersions were filtered through a 0.50 µm cellulose filter, and different secondary dopants (DMSO and EG) were added in different quantities (in wt%) (Table 1). The resulting solutions were stirred for 5 min using a magnetic stirrer. The dispersions were deposited on the substrates in 45 µL doses using a micropipette and were spin-coated with an initial drying stage at 500 rpm for 30 s, followed by an acceleration of 350 rpm/s to 3000 rpm for 60 s. Due to the small size of the substrate wafers, a build-up of solute due to the formation of a prominent edge bead could cause an uneven film surface. Therefore, the acceleration parameter played an important role in the overall uniformity of the film. In our trials, higher accelerations (≥350 rpm) were more successful in preventing the build-up of higher solute concentrations near the edge by overcoming the high surface energy of PEDOT particles. The samples were given around 6 h to dry (excluding transport between workstations) at 60 °C for the film to stabilize before the annealing process. The annealing was carried out under argon gas supplied at 1 sccm in a tube-type thermally insulated electric furnace (KTF0055N by Koyo Thermo Systems in Nara, Japan) at 140 °C for 15 min and allowed to cool down to room temperature. For morphological observations, the thickness, surface roughness, and surface profile of the samples were checked and measured using an SPM (SPM-9700 by Shimadzu Corporation in Kyoto, Japan) in the tapping mode. The grain radii and other dimensional information of the grains in each sample were measured and statistically analyzed using Gwyddion SPM analysis software (ver. 2.64) using a watershed model. For measuring the electrical characteristics, the samples were formed into rectangular shapes (10 mm × 2 mm) by removing excess parts from the spin-coated samples. The electrical contacts were made using carbon-pasted gold wires. The temperature dependence of the conductivity of these samples was tested with a standard DC four-probe method using a constant current supply (7651 Programmable DC Source by Yokogawa in Tokyo, Japan), and the voltage was measured using a Keysight Nanovolt meter 34420A for both cooling and heating cycles between 5 K and 296 K. The temperature of the films was controlled by placing the samples on a helium cooling head (4K Gifford-McMahon cryocooler RDK-101D by SHI Cryogenics Group in Kyoto, Japan) regulated by a cryogenic temperature controller (Model 335 by Lake Shore Cryotronics in Westerville, OH, USA) and a Cernox 1030SD temperature sensor (also by Lake Shore Cryotronics). The results were compared with previously obtained data measured on samples of PEDOT-P doped with 3 wt% of ethylene glycol (EG).

## 3. Results and Discussion

Thin films of PEDOT-F were prepared with the addition of 5 and 7 wt% DMSO (F5D and F7D) as they are the most commonly used concentrations typically for applications such as hybrid solar cells [8,9,10,11,12,13]. They were compared with samples of pristine PEDOT-F (F0) and PEDOT-P with 3% EG (P3EG). DMSO was chosen as a polar high boiling point solvent in this study due to its higher dielectric constant compared to EG. The expectation was that this would cause a larger screening effect between the counter ions (PSS) and the charge carriers (PEDOT) and minimize the Coulomb interaction between the PEDOT:PSS chains in PEDOT-F. After spin coating and annealing, the resultant thicknesses of the PEDOT-F and PEDOT-P samples on silicon substrates were roughly the same (Table 2). The dry film thickness ($h_{f}$) of a thin film after spin coating depends on the initial solute concentration ($C_{0}$), viscosity ($\eta_{0})$ and density (ρ) of the fluid, given by Meyerhofer’s equation:

(1) $$h_{f}={\left(\frac{3}{2}k\eta_{0}\right)}^{\frac{1}{3}}C_{0}{\left\{(1-C_{0})\rho \right\}}^{-\frac{1}{3}}\omega^{-\frac{1}{2}}$$

where *k* is a constant.

As we used the same preparation conditions (i.e., spin coating speeds and time) as some previous reports [22,23], the film thicknesses of the P3EG samples were comparable. The PEDOT-F films did not vary from the PEDOT-P samples by more than 15 nm. Considering PEDOT-P and PEDOT-F also have comparable densities and viscosities, it may be inferred that PEDOT-F likely has a similar solvent ratio and relative solute and solvent densities to PEDOT-P [32]. The roughness of the films was also similar to that of doped PEODT:PSS on ITO reported by Girtan et al. [33], validating the standard preparation of the films.

**Figure 2:**
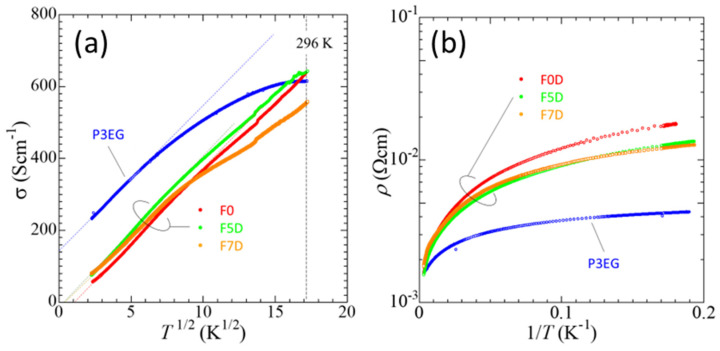
Temperature dependence of electrical characteristics of P3EG, F0, F5D, and F7d. (**a**) $\sigma \left(T\right)$ plotted on a *T*^1/2^ scale and (**b**) Arrhenius plot.

Figure 2a shows the temperature dependence of the electrical conductivity of a representative sample of the prepared PEDOT-F and PEDOT-P films. All conductivities showed a *T*^1/2^ dependence at low temperatures, as indicated by their linear dependences (dashed lines) on the horizontal scale of *T*^1/2^. This conductivity behavior conforms to a model of two-dimensional weak localization at low temperatures, which follows the relation,

(2) $$\sigma \left(T\right)=\sigma_{0}+mT^{1/2}+BT^{p/2}$$

where *m* and *B* are constants, and *p*~1 at low temperatures [34].

PEDOT:PSS is a complex organic compound comprised of both a highly conductive chain (PEDOT) that can have metal-like properties and an insulating polymer chain (PSS). In a dry thin-film, PEDOT:PSS grains are usually stacked as pancake-like microstructures that have PSS-rich regions in the contact areas [23]. Therefore, both metallic and insulating conduction models have to be considered when considering the charge mobility of PEDOT:PSS. The P3EG film showed a positive value of $\sigma_{0}$, which was obtained by making a linear extrapolation of $\sigma \left(T\right)$ toward 0 K. This behavior may indicate a possible disordered metallic state appearing even at 0 K where a finite high conductivity (~150 Scm^−1^) still exists. At higher temperatures, the dominating effect of this metallic state can explain the decrease in conductivity, possibly due to higher scattering of charge carriers. On the other hand, the F0, F5D, and F7D films all had negative values of $\sigma_{0}$. The negative $\sigma_{0}$ indicates that the origin of the *T*^1/2^ dependence of the conductivity in the F0, F5D, and F7D films cannot be explained by the weak localization model based on a metal state. The temperature dependence of the conductivity did not show activation-type behavior at all (Figure 2b), which indicates that these films did not have an intrinsic insulating state with an energy gap for charge transport. More studies of the charge transport mechanism, including the electronic state of PEDOT-F films, are needed. Figure 2a showed a trend where the conductivity of the F5D sample generally tended to exceed the conductivity of F0, whereas F7D was significantly poorer. A sharp bend occurs in the plot of F5D at about 265 K. This sudden dip in the temperature dependency curve is not present in the plots of the other samples and may have been caused by external factors. At 273 K, the conductivities for F0, F5D, and F7D samples were 610 S/cm, 630 S/cm, and 530 S/cm respectively.

**Figure 3:**
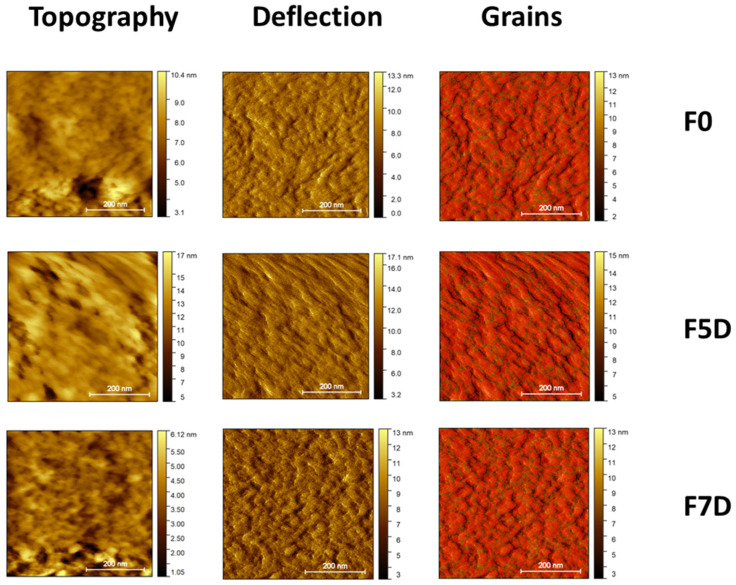
AFM topography, deflection, and a red mask layer representing grain boundaries calculated using a watershed algorithm of F0, F5D, and F7D films.

The addition of DMSO to pristine PEDOT-F seemed to change the surface texture of the films. AFM deflection images were used to create a map of probable grain boundaries on the surface using a watershed algorithm (Figure 3). Analysis of the top surface showed a difference in texture that was likely caused by a change in the overall alignment and stacking of PEDOT:PSS particles on the substrate. The grain maps of the AFM images were used to calculate the mean radius (R_m_) of the visible grains (Figure 3). The grains appear visibly elongated and oriented nearly flat on the surface of the F5D film. Measurements of the mean grain radii (the mean distance from the grain center of mass to its boundary) indicate that in a given 500 nm square area, F7D samples had 252 grains below 10 nm, while F5D and F0 films had 220 and 152 grains, respectively. On the other hand, for grains above 30 nm, F5D samples were the highest at 33, while F0 and F7D samples had 27 and 20, respectively.

**Figure 4:**
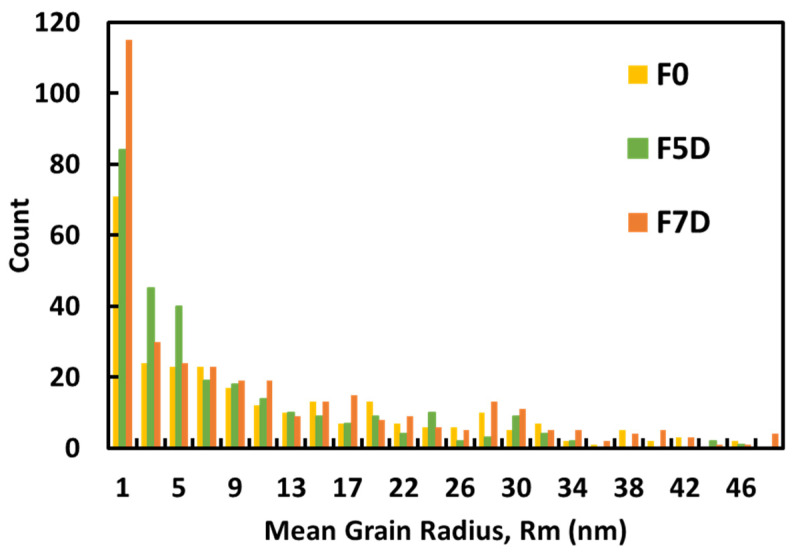
Distribution of the calculated mean radii of grains observed on the surface of F0, F5D, and F7D films.

To the best of our knowledge, the addition of polar solvents as secondary dopants to PEDOT-P has always led to a large increase in conductivity, typically two to three orders of magnitude. Takano et al. postulated that the increase in conductivity of PEDOT:PSS after adding solvents is induced by the formation and growth of PEDOT nanocrystals within the core region of the gel particle during the evaporation process when the film is being prepared [29]. Although PSS acts as the charge-balancing ion, it inhibits the growth of PEDOT nanocrystals due to the strong electrostatic coupling. Polar solvents help reduce these interactions through a shielding effect. This effect was further corroborated by Itoh et al., who observed this uncoupling phenomenon even at low concentrations of EG, while higher concentrations contributed to the growth of larger PEDOT nanocrystals [35]. Fernandez et al. [36] also reported that the size of PEDOT:PSS gel particles in a wet dispersion increased after the addition of DMSO. Therefore, the addition of solvents such as EG or DMSO forms larger PEDOT:PSS particles, which, after being stacked as a thin film during the coating process, increases the size of nanocrystals in the dried thin film and results in very high conductivities compared to pristine films. In the case of PEDOT-F, the pristine films themselves showed room temperature conductivities on par with those of doped PEDOT-P films. The change when adding secondary dopant, on the other hand, was only in the order of 10s of S/cm. In fact, the conductivity seemed to decrease with concentrations exceeding 5 wt%. It may be surmised that, unlike PEDOT-P, secondary doping does not play a role in untangling the PEDOT chains in PEDOT-F. Similarly, the overall size of individual PEDOT-F nanocrystals does not grow, unlike typically observed in PEDOT-P. Nevertheless, these smaller changes in conductivity are still above the margin of error and can probably be explained through the stacking of nanoparticle grains influenced by the concentration of secondary dopant in the solution. We hypothesize that the behavior of the DMSO-doped films can be explained in terms of the undulations observed on the thin film surface [26], which are indicative of the orientation of the underlying PEDOT:PSS microstructures (Figure 4).

**Figure 5:**
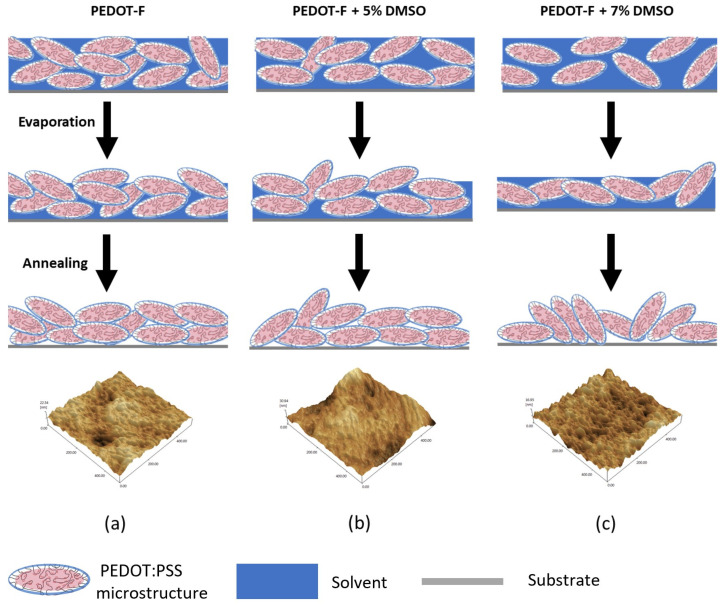
Three-dimensional AFM images (500 nm × 500 nm) of PEDOT:PSS thin films of (**a**) F0, (**b**) F5D, and (**c**) F7D with graphical representations of the formation of the underlying microstructure orientation given above. The light blue areas indicate PSS-rich regions, and the red ones indicate PEDOT-rich zones. The blue background indicates the available solvent in the film.

On dried films, the dimensions of the grains at the surface are projections of the overall stacking of individual PEDOT:PSS microstructures. The change in grain length suggests a shift in orientation or tilt of the elongated PEDOT:PSS particles. Roughness values, in terms of the arithmetic mean (R_a_) and root-mean-square (R_rms_) as measured by AFM, listed in Table 2 for all samples, show that the texture of F0D and F5D films are almost the same, while F7D films have a rougher texture. Combining this with the AFM image data, a surface with low roughness where grains visually seem more aligned would indicate that the individual particles were more horizontally oriented and stacked with respect to their longitudinal axes. Conversely, vertically oriented grains would present a rougher film with smaller visible grains. Adding 5 wt% DMSO, the top surface of the film exhibited slightly more elongated features compared with the pristine films. This resulted in a slightly smoother film. The morphology of the F7D film presented a texture with an increased density of sharper peaks and a corresponding increase in film roughness. Considering that all the DMSO would likely be removed by the annealing process and that the addition of DMSO would not increase the size of the PEDOT-F grains, we think that the higher roughness can only be explained by the aforementioned vertical stacking (Figure 5).

Due to the granular nature of intrinsically conducting polymers (ICPs) such as PEDOT:PSS, the charge transport is likely governed by both the intra-grain and inter-grain structures. The probable conduction pathways could be through (i) intrachain transport through conjugated PEDOT lamellae, (ii) inter-chain hops between adjacent PEDOT lamellae or (iii) interchain transport due to π–π stacking interactions [36,37]. Of all three interactions, the latter is the slowest and most resistive pathway, thus making it the rate-limiting step. A small decrease in the π–π stacking distance should have large changes in the overall conduction of interchain charge transfer and the conductivity of the film. The inter-grain conduction consequently depends on the morphology of the grains and is affected by the (i) elongation of the grain structure and (ii) stacking of the grains in relation to the direction of the current. In this study, the conductivities were measured parallel to the plane of the film. The quantity of the secondary dopant and its subsequent removal during the evaporation phase (during spin-coating) and annealing phase affect the final alignment of grains across the film (Figure 5). The favorable alignment of the PEDOT:PSS grains, namely one minimizing the overall density of PSS regions along the charge pathway, would play a factor in the conductivity of the film. Thus, the smoother F5D with more horizontally aligned grains had a higher conductivity compared to the pristine sample at most temperatures (Figure 5b). A larger amount of DMSO in solution increased the phase separation between PEDOT and PSS particles in the wet film [17]. Removal of this large amount of additive upon annealing allowed the thin film to shrink further, compacting the PEDOT:PSS grains in a more vertically aligned orientation, forcing more incoherent charge conduction to occur along the film (Figure 5c). Consequently, the conductivity of the F7D sample was lower by 85 S/cm compared with that of the pristine PEDOT-F. The change in the arrangement of PEDOT:PSS microstructures would also explain the visual contraction of the film due to the increased phase separation of PSS regions at the edges upon the addition of larger amounts of DMSO. Admittedly, we cannot explain the overall performance of the PEDOT-F solution as a whole. Further validation of the nanometer-scale changes in the grain structure using techniques such as X-ray diffraction (XRD) or grazing incidence wide-angle X-ray (GIWAXS) analysis is planned for future studies. PEDOT-F does not seem to require a secondary dopant to reach the same level of conductivity as PEDOT-P at room temperature. The pristine samples of PEDOT-F also seemed to have similar average grain sizes to those of the doped PEDOT-P film. While it is possible that PEDOT-F may already contain some form of secondary dopant, it exhibits very different temperature dependencies compared with conventionally doped PEDOT-P. This may mean that the fundamental charge transport mechanism in PEDOT:PSS has yet to be understood and may likely depend on factors that have not been addressed thus far.

## 4. Conclusions

Clevios F HC Solar is an aqueous dispersion of PEDOT:PSS nanoparticles that shows high conductivity without the addition of secondary dopants such as DMSO. This makes it an attractive candidate for the manufacture of Si/PEDOT:PSS hybrid solar cells. The temperature dependence of conductivity indicates that films made with this dispersion may not have an intrinsic insulating state with an energy gap for charge transport. The conductivity of F HC Solar seems to be tunable with the use of DMSO as a solvent. The addition of 5 wt% DMSO resulted in an improvement in conduction likely caused by a change in the morphology of the PEDOT:PSS grains from random orientations to a more uniform stacking of particles oriented longitudinally along the horizontal plane. Adding excess DMSO caused a change in the overall orientation of these microstructures due to a squeezing effect in the horizontal plane caused by the larger phase separation of PEDOT:PSS grains. Vertical stacks of PEDOT:PSS particles led to a greater amount of incoherent charge conduction along the film, reducing the conductivity by 85 S/cm. Controlling the shape and orientation of the PEDOT:PSS grains will lead to better control of the anisotropic conductivity of the films, depending on the application. For example, the appropriate addition of solvents or methods such as dip casting and flash annealing over a nanostructured substrate that can help orient the microstructures in a favorable direction may improve the conductivity even further. This can potentially impact the design of devices such as hybrid solar cells where the PEDOT film morphology affects the overall current density through the film, perpendicular to the film. Being able to tune and favorably orient the PEDOT grains in the direction of the current, especially on nanostructured substrates, will improve carrier mobility and, consequently, the efficiency of the cell.

## Figures and Tables

**Table 1:** Additives, amount in dispersion as a weight percent of the total mixture, and sample name.

Solution	Additive	wt%	Sample Name
PEDOT-P	Ethylene Glycol (EG)	3	P3EG
PEDOT-F	Pristine	0	F0
PEDOT-F	DMSO (D)	5	F5D
PEDOT-F	DMSO (D)	7	F7D

**Table 2:** Arithmetic mean and root mean square values of roughness (R_a_ and R_rms_) and thicknesses of PEDOT:PSS films.

Sample	R_a_ (nm)	R_rms_ (nm)	Thickness (nm)
P3EG	1.2	1.5	101
F0	1.4	2.0	115
F5D	1.3	1.8	107
F7D	2.7	3.5	109

## Data Availability

The data presented in this study are available on request from the corresponding author, Seiji Samukawa.
